# A Role for Tetracycline Selection in Recent Evolution of Agriculture-Associated *Clostridium difficile* PCR Ribotype 078

**DOI:** 10.1128/mBio.02790-18

**Published:** 2019-03-12

**Authors:** Kate E. Dingle, Xavier Didelot, T. Phuong Quan, David W. Eyre, Nicole Stoesser, Charis A. Marwick, John Coia, Derek Brown, Sarah Buchanan, Umer Z. Ijaz, Cosmika Goswami, Gill Douce, Warren N. Fawley, Mark H. Wilcox, Timothy E. A. Peto, A. Sarah Walker, Derrick W. Crook

**Affiliations:** a Nuffield Department of Clinical Medicine, John Radcliffe Hospital, University of Oxford, Oxford, United Kingdom; b National Institute for Health Research (NIHR) Oxford Biomedical Research Centre, John Radcliffe Hospital, Oxford, United Kingdom; c NIHR Oxford Health Protection Research Unit on Healthcare Associated Infection and Antimicrobial Resistance, John Radcliffe Hospital, Oxford University, Oxford, United Kingdom; d School of Life Sciences and Department of Statistics, University of Warwick, Coventry, United Kingdom; e Population Health Sciences, School of Medicine, University of Dundee, Scotland, United Kingdom; f Scottish Microbiology Reference Laboratories, Glasgow, United Kingdom; g University of Glasgow, Scotland, United Kingdom; h Department of Microbiology, Leeds General Infirmary, Leeds Teaching Hospitals, University of Leeds, Leeds, United Kingdom; Northern Arizona University

**Keywords:** *Clostridium difficile*, tetracycline resistance, whole-genome sequencing, phylogenetic analysis, emerging pathogen, PCR ribotype 078

## Abstract

The increasing clinical importance of human infections (frequently severe) caused by Clostridium difficile PCR ribotype 078 (RT078) was first reported in 2008. The severity of symptoms (mortality of ≤30%) and the higher proportion of infections among community and younger patients raised concerns. Farm animals, especially pigs, have been identified as RT078 reservoirs. We aimed to understand the recent changes in RT078 epidemiology by investigating a possible role for antimicrobial selection in its recent evolutionary history. Phylogenetic analysis of international RT078 genomes (isolates from 2006 to 2014, *n* = 400), using time-scaled, recombination-corrected, maximum likelihood phylogenies, revealed several recent clonal expansions. A common ancestor of each expansion had independently acquired a different allele of the tetracycline resistance gene *tetM*. Consequently, an unusually high proportion (76.5%) of RT078 genomes were *tetM* positive. Multiple additional tetracycline resistance determinants were also identified (including efflux pump *tet40*), frequently sharing a high level of nucleotide sequence identity (up to 100%) with sequences found in the pig pathogen Streptococcus suis and in other zoonotic pathogens such as Campylobacter jejuni and Campylobacter coli. Each RT078 *tetM* clonal expansion lacked geographic structure, indicating rapid, recent international spread. Resistance determinants for C. difficile infection-triggering antimicrobials, including fluoroquinolones and clindamycin, were comparatively rare in RT078. Tetracyclines are used intensively in agriculture; this selective pressure, plus rapid, international spread via the food chain, may explain the increased RT078 prevalence in humans. Our work indicates that the use of antimicrobials outside the health care environment has selected for resistant organisms, and in the case of RT078, has contributed to the emergence of a human pathogen.

## Abstract (precis)

Clostridium difficile PCR ribotype 078 (RT078) has multiple reservoirs; many are agricultural. Since 2005, this genotype has been increasingly associated with human infections in both clinical settings and the community. Investigations of RT078 whole-genome sequences revealed that tetracycline resistance had been acquired on multiple independent occasions. Phylogenetic analysis revealed a rapid, recent increase in numbers of closely related tetracycline-resistant RT078 (clonal expansions), suggesting that tetracycline selection has strongly influenced its recent evolutionary history. We demonstrate recent international spread of emergent, tetracycline-resistant RT078. A similar tetracycline-positive clonal expansion was also identified in unrelated nontoxigenic C. difficile, suggesting that this process may be widespread and may be independent of disease-causing ability. Resistance to typical C. difficile infection-associated antimicrobials (e.g., fluoroquinolones, clindamycin) occurred only sporadically within RT078. Selective pressure from tetracycline appears to be a key factor in the emergence of this human pathogen and the rapid international dissemination that followed, plausibly via the food chain.

## INTRODUCTION

Clostridium difficile infection (CDI) is a significant international challenge, affecting patients in community and health care environments worldwide (1–3). The severity of symptoms ranges from mild diarrhea to pseudomembranous colitis and toxic megacolon. Crude 30-day mortality in the United Kingdom is 16% (in a setting of endemicity) and can exceed 30% (4, 5), while it has been estimated that almost half a million CDIs caused 29,000 deaths in a single year in the United States (2).

The molecular epidemiology of CDI varies both temporally and geographically, frequently in response to local antimicrobial prescribing (2, 6–8). Clinically important outbreak-associated genotypes can emerge when the inherent resistance of C. difficile to cephalosporins (9) is supplemented with acquired resistance to certain high-risk antimicrobials, including clindamycin (10) and, more recently, fluoroquinolones. The latter contributed to the emergence of multiple phylogenetically unrelated outbreak-associated genotypes, including “hypervirulent” PCR ribotype 027 (11–13). However, the reason(s) for the changing prevalence of other clinically important C. difficile genotypes is frequently unknown (14).

The increased importance of C. difficile RT078 as a human pathogen was first reported in The Netherlands, with CDI cases rising from 3% to 13% during 2005 to 2008 (15). Around the same time, a 10-fold increase was noted in North America (16). Similar increases and occasional outbreaks were subsequently recorded throughout Europe (17–19), and the incidence of C. difficile RT078 infections has recently increased to 4.4%, 9.7%, and 8.1% of total CDI cases in North America, England, and Scotland, respectively (2, 20–22). Three distinctive features of RT078-associated CDI raise specific concerns, namely, increased severity of disease with the highest genotype-specific mortality rate (15, 23), a higher proportion of community-associated disease, and more infections in younger age groups than by other genotypes (2, 12, 15, 24).

The agricultural association of C. difficile RT078 is reflected in its isolation from sick and healthy animals (frequently pigs), bird droppings, vermin, and the farm environment (25–28). However, in common with many other toxin-producing C. difficile genotypes, ribotype RT078 can be carried asymptomatically by human infants and adults (29, 30). Isolates of RT078 recovered from humans and animals are genetically very similar and can be identical (30). This genotype has also been isolated from a variety of retail meat products, including pork, beef, and others (31, 32). Therefore, the natural reservoirs of RT078 support the hypothesis that humans become colonized via the food chain and/or the environment (25).

Whole-genome sequence data have been used to study the emergence and transmission of many bacterial pathogens. The international dissemination of hypervirulent fluoroquinolone-resistant C. difficile 027 was revealed in this way (12), and its rapid localized nosocomial transmission was demonstrated, as with other fluoroquinolone-resistant genotypes (8). Here, we used whole-genome sequencing and phylogenetic approaches to study the recent evolutionary history of C. difficile RT078 and to investigate the hypothesis that the recent clinical prominence of this genotype has been due to antimicrobial selection.

## RESULTS

The role of antimicrobial selection in the emergence of C. difficile RT078 was investigated using a large (*n* = 400), international collection of genomes from the United Kingdom, Europe, and North America (see Table S1 in the supplemental material) (8, 29, 30, 33, 34). Virtually all RT078 C. difficile strains share the same multilocus sequence type (ST), ST11 (35). However, this ST also includes the very closely related PCR ribotypes RT126, RT033, RT045, and RT066 (36) (the relationship to RT078 is shown phylogenetically in Fig. S1 in the supplemental material). Most of these ST11-associated PCR ribotypes (RT033, RT045, and RT066) are relatively rare clinically, with the possible exception of RT126 (18), which appears in our phylogeny to be descended from (rather than predating) RT078 (Fig. S1). Only one of the RT078 genomes included was a single-locus variant of ST11, namely, ST317. The overall proportion of RT078 within the collections from which the study genomes were sourced (see Materials and Methods) was 3% to 4%, because these data sets are dominated by RT027. The genomes studied are referred to here as RT078. The presence of determinants of resistance to tetracyclines (*tetM*), fluoroquinolones (*gyrA*/*B* substitutions), aminoglycosides [*aphA1* or AAC(6')-APH(2')], and clindamycin (*ermB*) within these genomes was assessed (Table 1 and 2).

**TABLE 1:** Antimicrobial resistance accessory genes used to search *C. difficile* whole-genome sequences^*a*^

Accessory gene	Reference sequence (GenBank)	Protein encoded	Antimicrobial resistance phenotype (predicted)
*tetM*^*d*^	NG_048243.1	Ribosomal protection protein	Tetracycline
*tetO*	AY394561.1	Ribosomal protection protein	Tetracycline
*tetW*	FR838948.1	Ribosomal protection protein	Tetracycline
*tetO*/*32*/*O*^*b*^	AJ295238.3	Ribosomal protection protein	Tetracycline
*tetB*(P)	NG_048319.1	Ribosomal protection protein	Tetracycline
*tet40*	JQ280445.2	Efflux pump	Tetracycline
*tetA*(P)	AB054980.1	Efflux pump	Tetracycline
*tetL*^*c*^	NG_048203.1	Efflux pump	Tetracycline
*ermB*^*e*^	HG002387.1	rRNA adenine N-6-methyltransferase	Macrolide-lincosamide-streptogramin B (MLS_B_) antibiotics, including clindamycin
*aphA1*^*f*^	M26832.1	Aminoglycoside 3′-phosphotransferase	Aminoglycoside (streptomycin)
AAC(6')-APH(2')	M13771.1	6'-N-Acetyltransferase and 2″-O-phosphotransferase activities, bifunctional	Most clinically important aminoglycosides,

a Additional classes of tetracycline resistance ribosomal protection proteins were searched for but not found, including those encoded by *tet*, *otrA*, *tetS*, *tetQ*, *tet36*, *tetT*, and *tet44* (75, 76). Additional tetracycline efflux pumps were searched for but not found, including those encoded by *tetA*, *tetB*, *tetC*, *tetD*, *tetE*, *tetG*, *tetH*, *tetJ*, *tetV*, *tetY*, *tetZ*, *tet30* (75).

b Ribosomal protection protein gene mosaic (77).

c Only found in ST54(012) (not RT078).

d *tetM* confers tetracycline resistance phenotype (30, 37, 38).

e Macrolide-lincosamide-streptogramin B (MLSB) resistance phenotype (conferred by the gene *ermB*) including clindamycin resistance, which has been associated with CDI outbreaks (10, 78–80).

f Aminoglycoside resistance as conferred by *aphA1* (30).

**TABLE 2:** Antimicrobial resistance housekeeping genes used to search *C. difficile* whole-genome sequences

Housekeeping gene	Nonsynonymous substitution	Protein modified	Antimicrobial resistance phenotype
*gyrA*^*a*^	T(82)I	DNA gyrase subunit A	Fluoroquinolones
*gyrB*^*a*^	D(426)N	DNA gyrase subunit B	Fluoroquinolones
*rpoB*^*b*^	R(505)K	β subunit RNA polymerase	Rifampicin

a Resistance to fluoroquinolones in C. difficile can be accurately predicted by the presence of the nonsynonymous mutations in the following genes: *gyrA* C(245)T [T(82)I] and *gyrB* G(1276)A and [D(426)N] (8 [Table S1], 81–83).

b The R(505)K substitution is the most frequently identified single substitution resulting in high-level (>32 mg/liter) resistance to rifampin (83, 84).

**FIG 1:**
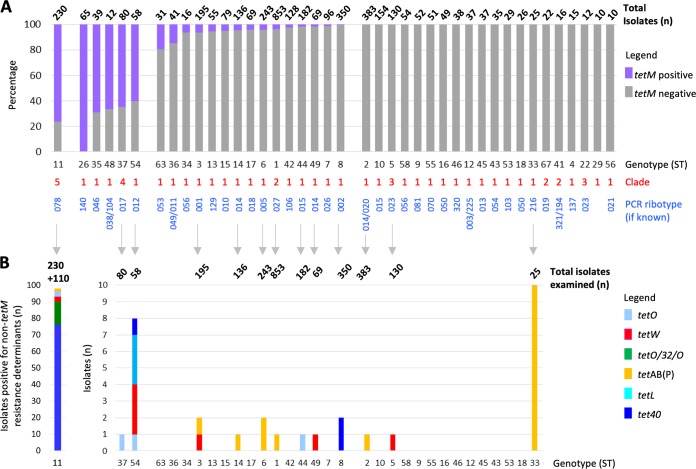
Prevalence of tetracycline resistance determinants in RT078 and other clinically relevant C. difficile genotypes. (A) Proportion (percentage) of each clinically important genotype that was positive for the ribosomal protection protein (RPP) gene *tetM*. Data are shown for genotypes having 10 genomes or more, from isolate collections representing Oxfordshire (EIA positives and negatives, infant and farm) and Leeds, North America, and Europe (Optimer clinical trial) (8, 29, 30, 33, 34). The total number of isolates of each genotype is shown above the bar. Clades are defined as described in reference 69. (B) Numbers of genomes in the collections described above which contained additional non-*tetM* tetracycline resistance determinants. For the ST11(078) genotype, the additional Scottish (*n* = 110) isolate collection was also included (indicated by “+110” above the bar at the left). Therefore, a total of 340 ST11(078) isolates were examined (the *n* = 230 described in the panel A legend above plus an additional *n* = 110 Scottish ST11s), the aim being to illustrate the overall prevalence of “non-*tetM*” tetracycline resistance determinants within this genotype.

10.1128/mBio.02790-18.1 FIG S1 United Kingdom-representative, time-scaled ST11(078) phylogeny. The phylogeny was constructed using the same genomes as were used for Fig. 1 but with an additional five genomes representing five closely related PCR ribotype reference isolates, all of which are ST11. Also, in contrast to Fig. 1, where the branches are colored to indicate geographic location, here the branch colors indicate the presence of tetracycline resistance determinants to highlight the associated independent clonal expansions. Download FIG S1, EPS file, 0.5 MB. Copyright © 2019 Dingle et al. 2019 Dingle et al. This content is distributed under the terms of the Creative Commons Attribution 4.0 International license.

10.1128/mBio.02790-18.5 TABLE S1 Details of the large international collection of genomes from the United Kingdom, Europe, and North America (*n* = 400) which was used to investigate the role of antimicrobial selection in the emergence of C. difficile RT078. The (co)occurrence of different antimicrobial resistance determinants in each genome is indicated, together with the SRA accession number. Download Table S1, XLSX file, 0.06 MB. Copyright © 2019 Dingle et al. 2019 Dingle et al. This content is distributed under the terms of the Creative Commons Attribution 4.0 International license.

### Prevalence of antimicrobial resistance determinants in C. difficile RT078.

*tetM*, the presence of which leads to a tetracycline-resistant phenotype (30, 37, 38), was by far the most prevalent antimicrobial resistance determinant in RT078, at 77.5% (148/191) among Oxfordshire and Leeds genomes, 76.4% (84/110) among Scottish genomes, and 75.0% (24/32) among North American and European genomes (Table S1). *gyrA*/*B* substitutions, which reduce susceptibility to fluoroquinolones, were less prevalent, at 13.0% (25/191) among Oxfordshire and Leeds RT078 genomes, 10.0% (11/110) among Scottish RT078 genomes, and 18.9% (6/32) in North American and European RT078 genomes. Similarly, *aphA1* (aminoglycoside resistance) was detected in 21.5% (41/191) of the Oxfordshire and Leeds genomes, 9.1% (10/110) of the Scottish genomes, and 40.6% (13/32) of the North American and European genomes. Finally, *ermB* (clindamycin resistance) occurred in 4.2% (8/191) of the Oxfordshire and Leeds genomes, 4.5% (5/110) of the Scottish genomes, and 18.8% (6/32) of the North American and European RT078 genomes. The co-occurrence of different resistance determinants (including tetracycline resistance determinants) within RT078 was found to be variable (Table S1).

### Prevalence of *tetM* in the clinical C. difficile population.

To determine whether the high level of *tetM* prevalence in RT078 was unusual among clinical C. difficile isolates, all available additional genomes of other genotypes (defined by ST/PCR ribotype) from the same isolate collections (except those comprising only RT078—the Scottish [first described here] and Dutch [30] isolates) were examined for the presence of *tetM* (Fig. 1A). The non-RT078 genotypes were described using the notation ST37(017) to indicate, for example, multilocus sequence type (MLST) 37 and the equivalent PCR ribotype (017).

Genotypes could be classified as (i) >60% *tetM* positive, (ii) >0% but <20% *tetM* positive (the majority being <5%), or (iii) *tetM* not detected (Fig. 1A). Non-RT078 genotypes were <20% *tetM* positive, with the notable exceptions of ST37(017) (52/80, 65.0%), ST54(012) (35/58, 60.0%), ST35(046) (27/39, 69.2%), and ST48 (8/12. 67.0%), plus nontoxigenic ST26(140) (65/65, 100%) (Fig. 1A). Therefore, at over 75%, RT078 was the most highly *tetM*-positive clinically relevant genotype.

### Prevalence of additional tetracycline resistance determinants in the clinical C. difficile population.

Genomes were assessed for the presence of additional tetracycline resistance determinants (Fig. 1B) (Table 1; see also Table S1). The tetracycline efflux pump gene *tet40* was present in 22.4% (76/340) RT078 (Fig. 1B; Oxfordshire, Leeds, Optimer Europe and North America, and Scotland genomes; *n* = 340, excluding previously published Dutch genomes [*n* = 60] because they had undergone prior analysis for tetracycline resistance [30]; however, for completeness, they are included in Table S1). Non-*tetM* ribosomal protection proteins were present in 6.5% (22/340) RT078 (Fig. 1B). In contrast to RT078, only one or two genomes of other ST/PCR ribotypes were positive for alternative tetracycline resistance determinants, except ST54(012) (*n* = 54), in which eight examples of four additional tetracycline resistance determinants were found, and ST33(216), which contained 10/25 (40%) *tetAB*(P) (Fig. 1B).

### United Kingdom-representative RT078 phylogeny.

**FIG 2:**
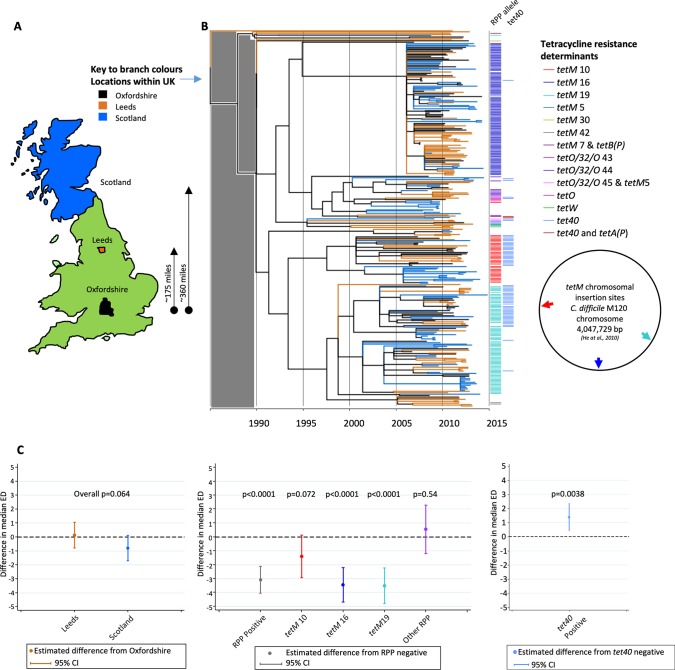
United Kingdom-representative, time-scaled RT078 phylogeny revealing a lack of geographic structure but strong structuring of tetracycline resistance. (A) Map showing the areas of the United Kingdom from which the RT078 C. difficile genomes were obtained. (B) Time-scaled ClonalFrameML phylogeny constructed using genomes from United Kingdom C. difficile isolates, comprising Oxfordshire (*n* = 78), Leeds (*n* = 104), and Scottish (*n* = 110) isolates. Branch colors, as defined for panel A, denote the location of each genome. Colored bars to the right of the phylogeny indicate the presence of tetracycline resistance determinants; ribosomal protection protein (RPP) allele sequences detected within each genome were assigned numbers to identify distinct nucleotide sequences of *tetM*, *tetO*/*32*/*O*, *tetO*, or *tetW*. To the right of the phylogeny, the chromosomal locations of the three most prevalent *tetM* alleles (designated *tetM* 10, 16, and 19) relative to the RT078 M120 genome (NCBI reference sequence NC_017174.1) are shown. All phylogenies included in this study are directly comparable post-1990, i.e., in the time frame of RT078 emergence; the gray shaded block over the region corresponding to the time period prior to that date indicates that region is not scaled identically and should not be used for comparisons. (C) The extent to which RT078 clonal expansions are associated with geographic structure and tetracycline resistance (ribosomal protection proteins and efflux pumps) was determined using two-sided quantile regression. (Left) Differences in median evolutionary distinctiveness scores compared to Oxfordshire samples. A lower evolutionary distinctiveness value indicates a larger proportion of close relatives in the tree. The *P* values indicate the overall significance of geographic location in the evolutionary distinctiveness score. (Center) Differences in median evolutionary distinctiveness scores for samples with ribosomal protection proteins detected compared to ribosomal protection protein-negative samples, overall and for each of the three putative *tetM*-associated clonal expansions. A lower evolutionary distinctiveness value indicates a larger proportion of close relatives in the tree. The *P* values indicate the significance of gene presence in the evolutionary distinctiveness score. (Right) Differences in median evolutionary distinctiveness scores for samples with tetracycline efflux pumps [*tet40* and *tetA*(*P*)] detected compared to efflux pump-negative samples. A lower evolutionary distinctiveness value indicates a larger proportion of close relatives in the tree. The *P* value indicates the significance of gene presence in the evolutionary distinctiveness score.

A United Kingdom-specific RT078 phylogeny was constructed using genomes from clinical infections in Oxfordshire (*n* = 78), the Leeds region (*n* = 104), and Scotland (*n* = 110) (Fig. 2A and B; see also Table S1). Annotation revealed minimal evidence of geographic structure, contrasting markedly with the highly structured distribution of *tetM* sequences (Fig. 2B and C) described in detail below (maximum likelihood [ML] phylogeny obtained before dating also supplied; see Fig. S2).

**FIG 3:**
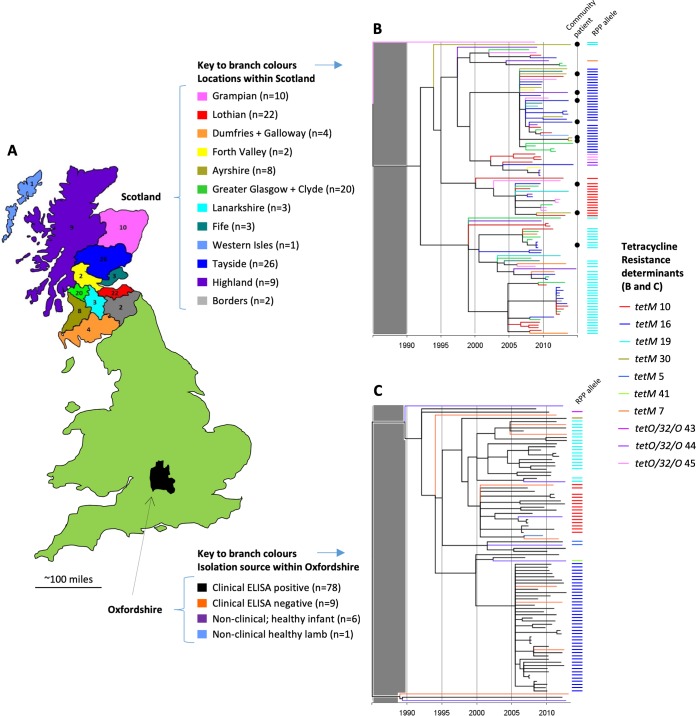
United Kingdom regional RT078 phylogenies; Scotland and Oxfordshire. (A) Map and legend indicating the regions of Scotland and Oxfordshire from which genomes originated. The Scottish regions correspond to administrative areas known as “health boards.” (B) Time-scaled RT078 phylogeny for Scotland. Branch colors are as described in the panel A legend. Colored bars to the right of the phylogeny denote the ribosomal protection protein (RPP) allele sequences detected within each genome (as described in the Fig. 2 legend), numbers being assigned to identify distinct nucleotide sequences of *tetM* or *tetO*/*32*/*O*. Isolates were cultured from human clinical samples received from both hospital and community patients, the latter being indicated by a black dot. The gray shaded block over the region corresponding to the period prior to 1990 indicates that the region is not scaled identically for different phylogenies and should not be used for comparisons. (C) Time-scaled RT078 phylogeny for Oxfordshire clinical and nonclinical isolates. Branch colors are as described in the panel A legend. Colored bars indicate ribosomal protection protein alleles as described above.

**FIG 4:**
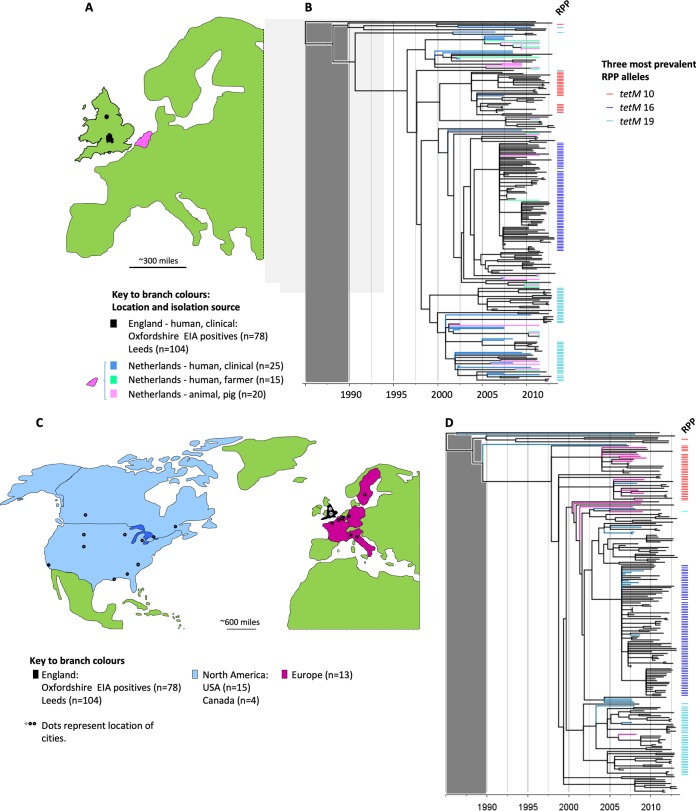
International phylogenies confirm that three major *tetM*-positive RT078 clades are present across continents. (A) Map of Western Europe. The regions of England and The Netherlands from which the genomes included in panel B originated are highlighted (in black and pink, respectively). (B) Time-scaled RT078 phylogeny constructed using genomes of clinical isolates from England (Oxfordshire and Leeds), supplemented with genomes from the Netherlands (human isolates [clinical or farmer] and pig isolates [30]). Branch colors are as described for the map in panel A. The presence of the three predominant ribosomal protection protein (RPP) *tetM* alleles (*tetM* 10, 16, and 19) is indicated by the colored bars to the right of the tree. The gray shaded block over the region corresponding to the period prior to 1990 indicates that the region is not scaled identically for different phylogenies and should not be used for comparisons. (C) Map highlighting the regions in North America and Western Europe from which the genomes included in panel D originated. (D) Time-scaled RT078 phylogeny constructed using genomes of clinical isolates from England supplemented with clinical isolates from North America and Europe (distinct from the isolates used as described for panel B) from two clinical trials of the drug fidaxomicin (Table S1) (33, 34).

**FIG 5:**
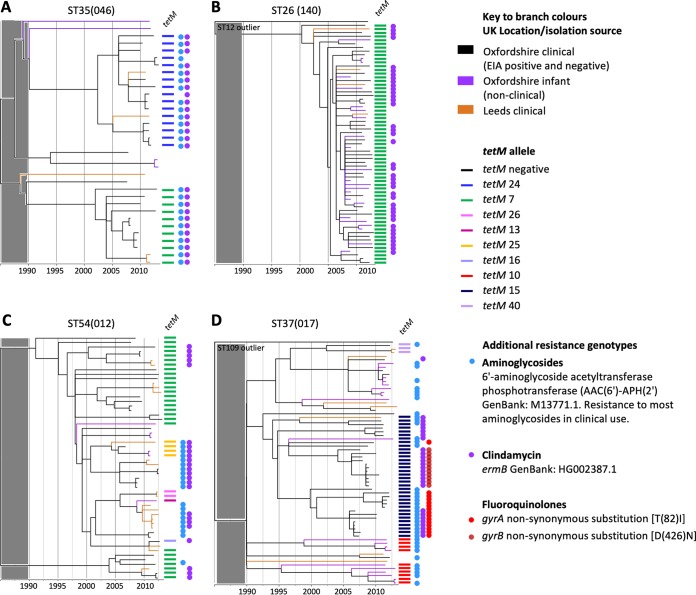
Phylogenetic analysis of additional *tetM*-positive C. difficile genotypes. Time-scaled phylogenies were constructed representing four non-RT078 genotypes with >60% *tetM* prevalence as follows: (A) ST35(046), (B) ST26(140), (C) ST54(012), and (D) ST37(017). In the phylogenies shown in panels B and D, a single closely related genome of a distinct genotype (ST12 and ST109, respectively) was included to ensure that the tree was rooted pre-1990 and that the four phylogenies could therefore be compared post-1990. The gray shaded block over the region corresponding to the period prior to 1990 indicates that the region is not scaled identically for different phylogenies and should not be used for comparisons. Genomes were from Oxfordshire (clinical EIA positives and negatives plus nonclinical, healthy infants) and Leeds (clinical isolates); branch colors indicate location/isolation source as described above. Colored bars to the right of each phylogeny indicate the presence of tetracycline resistance determinants. Colored dots represent additional genetic determinants identified as conferring resistance to fluoroquinolones, rifampin, clindamycin, and aminoglycosides (Table 1 and 2) and are shown where five or more positive genomes were identified per genotype.

10.1128/mBio.02790-18.2 FIG S2 The nine ML phylogenies obtained before dating for each of the time scaled phylogenies shown in the main figures (Fig. 2, 3, 4, and 5). Download FIG S2, EPS file, 0.4 MB. Copyright © 2019 Dingle et al. 2019 Dingle et al. This content is distributed under the terms of the Creative Commons Attribution 4.0 International license.

Prior to annotation, distinct *tetM* allele sequences were assigned a number (available at https://pubmlst.org/bigsdb?db=pubmlst_cdifficile_seqdef&page=downloadAlleles). Among the *tetM*-positive United Kingdom RT078 genomes, the following three *tetM* alleles predominated: *tetM* 10 (36/292, 12.3%), *tetM* 16 (101/292, 34.6%), and *tetM* 19 (78/292, 26.7%) (Fig. 2B). Colored bars (Fig. 2B) (or branches in Fig. S1) were used to identify distinct *tetM* alleles. Each of *tetM* alleles 10, 16, and 19 were carried by closely related, Tn*916*-like conjugative transposons (well-established Gram-positive *tetM*-carrying mobile elements) (37). Independent acquisition events, estimated from the phylogeny to have occurred between 1995 and 2006, were suggested by their unique chromosomal insertion sites (Fig. 2B, circular map) and by the level of nucleotide sequence identity across the Tn*916*-like elements on which they were carried (87% to 100%, depending on the region compared) (Fig. S3). Acquisition of *tetM* 16 or *tetM* 19 was associated with significantly shorter branch lengths (confirmed by median evolutionary distinctiveness [ED] scores of 3.78 and 3.58, respectively, versus 7.22 for branches representing genomes lacking a ribosomal protection protein gene; *P* < 0.001). This observation is consistent with the presence of clonal expansion in response to tetracycline-associated selection pressure (Fig. 2B and C); significantly lower ED scores indicate unexpectedly short branches (39). It is possible that, for a given branch, there could be some genetic change other than *tetM* that was the cause of the clonal expansion, but since the same pattern was observed on several independent branches where *tetM* was acquired, each time within a different Tn*916* variant (Fig. S3), it seems very likely that this underlies the clonal expansion. The acquisition of efflux pump *tet40* on its own was not associated with clonal expansion, with only a slightly higher median evolutionary distinctiveness score than was calculated for *tet40* absence (Fig. 2C).

10.1128/mBio.02790-18.3 FIG S3 Comparison of two Tn*916*-like elements carrying distinct *tetM* alleles (designated alleles 10 and 16). Comparisons were generated using the Artemis Comparison tool (ACT) (available at https://www.sanger.ac.uk/science/tools/artemis-comparison-tool-act). The central blue area joins the Tn*916*-like elements, and their levels (percentages) of nucleotide sequence identity are shown in white. The *tetM* and transposon integrase genes are shown in red. The absence of identity outside the (blue) sequence of the mobile elements highlights their insertion into completely unrelated regions of the chromosome, i.e., independent acquisition events. Download FIG S3, EPS file, 0.6 MB. Copyright © 2019 Dingle et al. 2019 Dingle et al. This content is distributed under the terms of the Creative Commons Attribution 4.0 International license.

The same phylogeny was annotated for the presence of additional resistance determinants (conferring aminoglycoside, fluoroquinolone, or clindamycin resistance; Fig. S4A to C), but no evidence of associated clonal expansions occurring independently of the *tetM*-associated expansions was found (Fig. S4D to F).

10.1128/mBio.02790-18.4 FIG S4 United Kingdom-representative, time-scaled ST11(078) phylogeny. The phylogeny is the same as that in Fig. 1; however, here the branch colors indicate the presence of the following antimicrobial resistance determinants. (A) Aminoglycoside resistance; *aphA1* (aminoglycoside 3′-phosphotransferase) associated with resistance to streptomycin. (B) Fluoroquinolone resistance conferred by nonsynonymous *gyrA* or *gyrB* substitutions. (C) Clindamycin resistance; *ermB*. (D to F) Data represent the extent to which 078 clonal expansions are associated with antimicrobial resistance *aphA1* determinants, *gyrA*/*B* substitutions, and *ermB*, using two-sided quantile regression. Differences in median ED (evolutionary distinctiveness) scores (39) for isolates with the resistance determinant compared to isolates without are indicated. A lower ED value indicates a larger proportion of close relatives in the tree. The *P* value represents the significance of the presence of the resistance determinant with respect to the ED score. Download FIG S4, EPS file, 0.2 MB. Copyright © 2019 Dingle et al. 2019 Dingle et al. This content is distributed under the terms of the Creative Commons Attribution 4.0 International license.

### RT078 phylogenies representing United Kingdom regions.

Separate phylogenies were constructed to examine the detailed evolutionary history of RT078 within two of the geographic regions represented in the United Kingdom phylogeny. The two regions were Scotland (population, 5.295 million; area, 30,918 square miles) (Fig. 3A and B) and Oxfordshire (population, 655,000; area, 1,006 square miles) (Fig. 3A and C) (ML phylogeny obtained before dating also shown; Fig. S2).

The branches of the Scottish phylogeny (*n* = 110 genomes; Fig. 3B) were colored to represent geographic regions (administrative areas, or “health boards”; Fig. 3A), thus increasing the level of geographic discrimination. As described above, geographic structure was absent, with health care-associated and community isolates intermingling (Fig. 3B, dots), but the distribution of the *tetM* alleles 10, 16, and 19 within the phylogeny was highly structured.

The Oxfordshire regional phylogeny (*n* = 94 genomes, Fig. 3C) represented a more densely sampled, smaller geographic area (Fig. 3A). Here, the enzyme immunoassay (EIA)-positive C. difficile clinical isolate genomes (*n* = 78; Fig. 2B) were supplemented with EIA-negative clinical isolates (*n* = 9) (i.e., isolates from patients with diarrhea but without evidence of toxin production, suggesting that C. difficile was colonizing the patient rather than causing disease) and nonclinical isolates from healthy infants (*n* = 6) (29) and a lamb (*n* = 1) (Table S1). All genomes were pathogenicity locus (PaLoc) (i.e., toxin A and B encoding sequence) positive (40). This regional phylogeny also lacked structure according to location or isolation source, but it was again structured according to *tetM* allele (Fig. 3C).

### International phylogenies confirmed that three *tetM*-positive RT078 clades are present across continents.

Two international RT078 phylogenies were constructed using genomes from clinical infections in England (Oxfordshire [*n* = 78] and Leeds [*n* = 104]) supplemented first with clinical and nonclinical isolates from The Netherlands (30) (Table S1; clinical isolates from humans [*n* = 25] and isolates collected from farmers [*n* = 15] and pigs [*n* = 20]) (Fig. 4A and B), and second with genomes from clinical infections in North America (Table S1; United States [*n* = 15], Canada [*n* = 4]) and Europe (five countries [*n* = 13]) (Fig. 4C and D). Once again, structure according to geography was absent, but structuring by *tetM* allele (Fig. 4B and D) was clear (ML phylogeny obtained before dating also shown; see Fig. S2).

### Tetracycline selection in other C. difficile genotypes.

Over 60% of genomes belonging to each of five non-RT078 genotypes were *tetM* positive (Fig. 1A). Four of these were investigated phylogenetically, namely, ST37(017), ST54(012), ST35(046), and nontoxigenic ST26(140) (8, 29) (Table S2), but not ST48(038/104), as only 12 genomes were available. Branches were colored according to isolation source and geography as described above, and *tetM* alleles are indicated by colored bars (Fig. 5). As described above for RT078, additional resistance determinants (Table 1 and 2) were also highlighted (when present in five genomes or more) to reveal the possible impact of selection by other antimicrobials (Fig. 5, colored dots) (ML phylogeny obtained before dating also shown; Fig. S2).

10.1128/mBio.02790-18.6 TABLE S2 Details of the genomes used to construct the phylogenies for *tetM*-positive non-RT078 genotypes (Fig. 5). Genomes (each over 60% *tetM* positive) were investigated phylogenetically for the following four non-RT078 genotypes: ST37(017), ST54(012), ST35(046), and nontoxigenic ST26(140). Download Table S2, XLSX file, 0.04 MB. Copyright © 2019 Dingle et al. 2019 Dingle et al. This content is distributed under the terms of the Creative Commons Attribution 4.0 International license.

A number of recent *tetM* acquisition events were obvious (Fig. 5). These were followed by possible clonal expansions, most notably within genotypes ST35(046) and (nontoxigenic) ST26(140) (Fig. 5A and B). Clonal expansion was particularly marked in ST26(140), where all genomes were *tetM* positive, and clonal expansion occurred in the absence of disease-causing ability, this genotype being nontoxigenic (lacking the pathogenicity locus [PaLoc] in all genomes [40]). With the exception of ST35(046), where aminoglycoside and clindamycin resistance determinants colocalized with *tetM* (Fig. 5A), and the fluoroquinolone-resistant region of the ST37(017) phylogeny (Fig. 5D) (8), there was no clear evidence of the clonal expansions which had followed the acquisition of the non-*tetM* antimicrobial resistance determinants. In common with RT078, all four phylogenies (Fig. 5) lacked geographic structure, with the exception of the fluoroquinolone resistance region of the ST37(017) phylogeny (Fig. 5D) (8).

### Sequences of RT078 tetracycline resistance determinants support the hypothesis of its zoonotic origin.

The *tetM* sequences in C. difficile described here are typical of many Gram-positive species, including established zoonotic species. For example, the RT078 *tetM* 10 allele shared 100% nucleotide sequence identity with *tetM* genes of Streptococcus agalactiae, Enterococcus faecalis, Escherichia coli, and Streptococcus pneumoniae and 99% nucleotide sequence identity with Streptococcus suis (a pathogen of pigs transmitted zoonotically to humans [41, 42]). Identical *tetM* 10 sequences have also been found in Gram-negative bacterial species, including Escherichia coli. The RT078 *tetM* 16 allele shared >97% nucleotide sequence identity with *tetM* in *Enterococcus* species (mostly E. faecalis and E. faecium), followed by *Staphylococcus* and *Streptococcus* species, including S. suis.

Other RT078 tetracycline resistance determinants were also identical or were very closely related to those found in bacteria with an agricultural association which may be zoonotic. For example, RT078 *tet40* sequences shared 99% to 100% identity with Streptococcus suis *tet40* (GenBank accession no. KC790465.1) and the RT078 *tetO* sequences shared over 99% nucleotide sequence identity with Campylobacter jejuni, Campylobacter coli, and S. suis *tetO* sequences. In addition, the RT078 *tetO*/*32*/*O* mosaic sequence shared 99% identity with the sequence found in the S. suis genome.

## DISCUSSION

Our time-scaled phylogenies revealed geographically unstructured, parallel *tetM*-associated RT078 clonal expansions, dating from around the year 2000 (Fig. 2B, 3B and C, and 4B and D). These findings are consistent with an evolutionary response to tetracycline selective pressure, within the milieu of tetracycline-resistance determinants, during the time frame of increasing numbers of RT078-associated clinical cases (15, 18–20, 22). The results from our use of whole-genome sequence-based phylogenies explained the prior observation (using multilocus variable-number tandem-repeat analysis [MLVA] [38]) that the majority (85%) of human and porcine RT078 genomes are genetically related, irrespective of the European country of origin, since we showed that most RT078 genomes are recent descendants from one of three distinct (but closely related and now internationally disseminated) *tetM*-positive ancestral RT078 genomes (Fig. 2 to 4).

Tetracyclines were initially introduced around 60 years ago in both clinical and veterinary settings. However, following the emergence of resistance, they were largely replaced in human medicine by fluoroquinolones (43). Consequently, by 2010 to 2013, tetracyclines represented a total of <18% of the antibiotics consumed by patients in England, and most (92%) were prescribed in the community by general practitioners (44). Over the time period relevant to this study, tetracyclines were most commonly used for the treatment of acne and chlamydial sexually transmitted diseases. It is implausible that such prescribing in teenagers and young adults provided extensive selection pressure for C. difficile, given that healthy individuals living in the community have very low rates of colonization by these bacteria (45).

In contrast to their use in human medicine, tetracyclines remain the most widely used antimicrobial for the treatment, control, and prevention of infections in animals (46). In addition, their use for growth promotion (in subtherapeutic doses) continues around the world, if not overtly, under the guise of disease prevention. This is the case despite the fact that growth promotion is banned in Europe (47) and that claims of growth promotion have been voluntarily removed by drug companies in the United States at the request of the FDA (January 2018). During 2015, 6,880 metric tons of tetracyclines were sold in the United States (48) (representing a 31% increase from 2009), compared to 166 tons in the United Kingdom (49). The extent of agricultural tetracycline use, the prevalence of RT078 in animals used for food (25, 26, 31, 32, 38), and the time frame of RT078 emergence all implicate tetracycline use in agriculture as a plausible source of selective pressure. The global food chain, including, for example, regionally concentrated livestock production followed by widespread distribution of meat products, represents an obvious route for rapid RT078 dissemination (food and livestock RT078 genomes/isolates are listed in references 25 and 50), which would be consistent with our phylogenies (Fig. 2 and 4). However, indirect transmission from the agricultural environment to humans via contaminated water or vegetables (51) is also a possibility. Our report provides evidence of a plausible agricultural link underlying the emergence of RT078 by presenting its recent evolutionary history with respect to the acquisition of antimicrobial resistance.

The absence of geographic structure within our RT078 phylogenies is consistent with its rapid international spread (Fig. 4) as described previously (38, 50) and with the lack of large-scale, localized nosocomial RT078 outbreaks (Fig. 2B, 3B and C, and 4B and D). Among other genotypes, such outbreaks have been associated with extensive prescribing of, and resistance to, high-risk antimicrobials such as clindamycin, cephalosporins, and fluoroquinolones. In our study, large-scale clonal expansions were not associated with fluoroquinolone or clindamycin resistance in RT078 genomes (see Fig. S4 in the supplemental material). Equivalent analyses for cephalosporins cannot be performed because the genetic mechanism(s) of cephalosporin resistance in C. difficile has yet to be defined, and, although MICs can vary, C. difficile has typically been considered inherently cephalosporin resistant, irrespective of genotype (9). The international spread of RT078 indicates that changes in antimicrobial resistance phenotypes could potentially have an impact at any location, depending on local prescribing practices. Consequently, RT126, a frequently isolated fluoroquinolone-resistant descendant of RT078 (Fig. S1, phylogenetic context), which is prevalent in Italian clinical settings (52, 53), is of particular concern, as is the epidemic multidrug-resistant RT078 observed in Spanish swine (54).

Tetracyclines (such as doxycycline) are associated with a lower risk of CDI in humans (55) than has been established for many antimicrobials. It has been proposed (55) that the use of tetracyclines as an alternative to riskier antimicrobials such as fluoroquinolones and clindamycin, whenever appropriate, may decrease CDI associated with antibiotic use. However, the emergence of tetracycline-resistant C. difficile genotypes such as RT078 and others (Fig. 5) may require this approach to be informed by resistance data for the infecting strain, to avoid triggering tetracycline-resistant CDI. Tetracyclines may be unrecognized as a potential CDI risk factor, since resistance emerged relatively recently (with respect to RT078) and is less common at the population level (Fig. 1A); clindamycin-resistant and fluoroquinolone-resistant strains in particular predominate under outbreak conditions (10–12).

The identification of widespread tetracycline resistance (>60% *tetM* positive) in only five C. difficile genotypes in addition to 078 (Fig. 1A) is consistent with previous reports (17, 56, 57). Phylogenetic analysis showed that ST35(046) contained two plausible *tetM*-associated clonal expansions (Fig. 5A), but the relatively small numbers precluded quantitative evolutionary distinctiveness analysis. Like RT078, ST35(046) has been found in pigs and has caused human outbreaks of CDI (58). This genotype also illustrates the possibility that selection by one antimicrobial can drive the acquisition of further, linked resistance genes, as almost every *tetM*-positive ST35(046) genome was also positive for clindamycin and aminoglycoside resistance determinants (Fig. 5A). Nontoxigenic ST26(140) contained *tetM* in every genome examined, suggesting stable integration predating a recent clonal expansion (Fig. 5B) concurrent with that of RT078. ST26(140) therefore illustrates the possible consequences of tetracycline selection in a harmless commensal organism, confirming that tetracycline selection alone may have been sufficient to drive the emergence of RT078.

The hypothesis that RT078 has an agricultural origin is further supported by the observation that RT078 shares many resistance determinants with zoonotic pathogens such as Streptococcus suis, Campylobacter jejuni, and C. coli, suggesting a common reservoir (see Results). Quantitative analysis (Fig. 2C; see also Fig. S4E and F) confirmed that *tetM* was associated with RT078 clonal expansions. Although widespread in RT078, the tetracycline efflux pump *tet40* did not show such an association on its own (Fig. 2B and C). Efflux pumps often confer a low-level-resistance phenotype, assisting bacterial survival at sublethal concentrations of antimicrobials (for example, *tetK* in livestock-associated methicillin-resistant Streptococcus aureus [LA-MRSA] CC398 [59]). They thereby function in promoting the acquisition of further high-level-resistance determinants, such as *tetM*. The parallels between RT078 and zoonotic Streptococcus suis also extend to their epidemiology. S. suis is a globally distributed emergent pathogen of humans (42), commonly isolated from pigs. Geographic clustering of subpopulations is absent (41), and S. suis has exhibited rapid, recent increases in tetracycline resistance (59). The emergence of human pathogens, coincident with tetracycline resistance acquisition, has also been noted among other bacterial species. Tetracycline resistance in group B streptococci may have contributed to its emergence as a leading cause of human neonatal infections (60). In a study examining LA-MRSA S. aureus CC398 isolates, almost all were found to be *tetM* positive, and many were also found to carry the *tetK* (efflux pump) gene (61).

Although multiple lines of evidence indicate a role for tetracycline selection in the recent evolutionary history of RT078, the possibility exists that further lineage-specific genetic changes (unrelated to tetracycline resistance) contributed to its *tetM*-associated clonal expansions (Fig. 2 and 4; see also Fig. S2). A recently proposed hypothesis is that an enhanced ability to metabolize the disaccharide trehalose (conferred by a specific four-gene chromosomal insertion) helped to drive the emergence of C. difficile RT078 in humans (62) due to the introduction of trehalose as a food additive. The authors of that study identified the same gene cluster in closely related non-078 clade 5 (clades defined as described in reference 35) PCR ribotypes (033, 045, 066, and 126). Therefore, the possibility that the insertion occurs throughout clade 5 or, indeed, among the other four C. difficile clades was not excluded. The trehalose and tetracycline hypotheses are not mutually exclusive. However, the available evidence suggests that tetracycline resistance driven by *tetM* acquisition remains the most plausible available explanation for the recent clonal expansions observed in RT078.

The role of selection by antimicrobials other than tetracycline (fluoroquinolones, clindamycin, aminoglycosides) was investigated (Fig. S4), and a small potential contribution by aminoglycosides (also used in animal production) was indicated by the presence of the *aphA1* resistance gene in a minority of RT078 genomes (Fig. S4A). However, *aphA1* could not be assessed independently of *tetM* (Fig. S1) because the two genes colocalized. Further work would be required to compare the total gene content of *tetM*-positive RT078 isolates with that of older *tetM*-negative isolates to identify further potentially relevant genetic differences that could explain the clonal expansions. The identification of *tetM*-associated clonal expansions in genetically divergent C. difficile genotypes, ST35(046) (together with clindamycin and aminoglycoside resistance determinants; Fig. 5A) and nontoxigenic ST26(140) (Fig. 5B), serves to further highlight *tetM* as a factor common to recent clonal expansions within distinct C. difficile genetic backgrounds. To further confirm the zoonotic origin of RT078 and the link to agricultural tetracycline use, large-scale, parallel data showing changing tetracycline use over time and concurrent RT078 isolates from clinical cases and farm animals would be required. Although it would be challenging to source both usage data and corresponding isolate collections retrospectively, evidence of phylogenetic coclustering of human and animal RT078 genomes has been provided both nationally and internationally (30, 50), using collections assembled as available from other studies and reference laboratories.

In summary, numerous lines of evidence described in this and prior work (25, 30, 50) support the hypothesis that tetracycline use in agriculture has provided recent selection pressure which has impacted on the evolution of tetracycline-resistant RT078. This in turn supports the hypothesis (first proposed in 2012 [25]) that humans become colonized by RT078 via the food chain and/or the environment. Recent studies using whole-genome sequencing of 65 Dutch RT078 isolates and 248 international RT078 isolates (30, 50) provided data consistent with the rapid spread of RT078 both internationally and between animals and humans. Furthermore, a range of tetracycline-resistant determinants were described in both human and animal RT078 populations, including *tetM*, *tet40*, *tet32*, *tet44*, and *tetO* (30, 50). Our findings independently confirm and extend this work, since we demonstrate at least three independent clonal expansions of RT078, with rapid international spread, following unconnected *tetM* acquisition events (*tetM* carried on distinct Tn*916* variants inserted into distinct chromosomal locations; Fig. S3) which occurred in well-separated regions of the RT078 phylogeny (Fig. 2 to 4). We also show that the RT078 genome is almost unique within the C. difficile population as a whole, in terms of the diversity of its tetracycline resistance determinants (Fig. 1). The major C. difficile RT078 transmission routes to humans are consequently more likely to be related to agriculture and international food chains than nosocomial. Our findings add to the body of evidence (50) supporting initiatives such as “One Health” (63). Our findings strongly suggest that the use of tetracycline outside the health care environment has impacted several C. difficile genotypes, most strikingly RT078, and therefore has not only selected for resistant organisms but also contributed to the emergence of this species as a human pathogen.

## MATERIALS AND METHODS

### C. difficile whole-genome sequences.

C. difficile genomes derived from isolates of either RT078 or ST11 (*n* = 400) were sourced from several published collections (8, 29, 30, 33, 34), as well as from an unpublished Scottish collection and the Oxford University farm, Wytham, United Kingdom (see items i to v below and Table S1 in the supplemental material). Each isolate was obtained from a distinct sample. EIA-negative isolates were inferred to be toxigenic or nontoxigenic, depending on the presence/absence of the toxin-encoding pathogenicity locus (PaLoc) (40) (Tables S1 and S2). The complete collections described in sections i, iii, and iv below have been published previously (8, 29, 30).

### (i) Clinical C. difficile: Oxfordshire and Leeds, United Kingdom.

Genomes were available for 87 RT078 or ST11 C. difficile isolates cultured from symptomatic Oxfordshire patients by the Clinical Microbiology Laboratory, Oxford University Hospitals NHS Trust, Oxford, United Kingdom, between September 2006 and April 2013 (8). Seventy-eight isolates were derived from toxin enzyme immunoassay (EIA)-positive stools (initially [i.e., until April 2012] by the use of the Meridian Premier Toxins A&B Enzyme Immunoassay [Meridian Bioscience Europe, Milan, Italy] and subsequently by the use of the TechLab Tox A/B II assay [TechLab Inc., Blacksburg, VA, USA]) and 9 from EIA-negative but glutamate dehydrogenase (GDH)-positive samples (by the use of Premier C. difficile GDH EIA [Meridian Bioscience Europe, Milan, Italy]) (8). Genomes were also available for 104 RT078 C. difficile-positive stool samples (identified using cytotoxin testing) obtained from routinely examined diarrheal fecal samples at the Leeds Teaching Hospitals NHS Trust between August 2010 and April 2013 (Table S1).

### (ii) Clinical C. difficile: Scotland, United Kingdom.

The isolates from Scotland, United Kingdom, included 109 isolates of RT078 and 1 closely related RT066 isolate (Table S1). These isolates form part of a collection stored at the Scottish Microbiology Reference Laboratory (Glasgow). Cultures are provided by Scottish regional NHS Healthcare Boards (located per the map shown in Fig. 3A) in the event of a severe/fatal case, a suspected outbreak, or a suspected ribotype 027 infection. In addition, each Health Board provides a fixed number of samples based on the rates of infection/population. This allows surveillance of prevalent circulating strains to be assessed. RT078 isolates for this study were selected based on the ribotypes from samples referred to the Reference Laboratory between November 2007 and October 2014 and with the aim of providing the widest temporal and geographical representation. Locally, positive fecal stool samples were identified prior to 2009 using a toxin-specific EIA (or cell cytotoxicity) and post-2009 by the use of a two-step algorithm requiring GDH detection, followed by toxin assessment. These samples were from patients located in health care (*n* = 99) and community (*n* = 10) settings (unassigned *n* = 1) in 12 of 14 Health Boards (there were no relevant samples from 2 small-island Health Boards [not shown in the map in Fig. 3A]).

### (iii) Clinical C. difficile: North America and Europe.

Thirty-two ST11 genomes from North American (Canada, *n* = 4; United States, *n* = 15) and European (*n* = 13) C. difficile isolates cultured from clinical infections between November 2006 and June 2009 were available from a variety of locations from two clinical trials of fidaxomicin (Table S1, showing city and country [33, 34]). Previously published RT078 genomes from human clinical cases (*n* = 25; 2002 to 2011) in The Netherlands were also included (30) (Table S1).

### (iv) Nonclinical C. difficile.

Six ST11 isolates were cultured from the stools of healthy, asymptomatic Oxfordshire infants (29) between April and October 2012 (Table S1). A single ST11 isolate was isolated from a lamb at Oxford University Farm, Oxfordshire, United Kingdom, in June 2009. Previously published C. difficile RT078 genomes from farmers (*n* = 15; 2009 to 2011) and pigs (*n* = 20; 2008 to 2011) in The Netherlands were included (30) (Table S1).

### (v) PCR ribotype reference isolates.

For additional context, five PCR ribotype reference C. difficile genomes representing RT078, RT126, RT033, RT045, and RT066 were included, all of which are genetically very closely related, sharing the same multilocus sequence type, ST11 (see Table S1 and Fig. S1 in the supplemental material).

### (vi) Four additional genotypes.

Four additional C. difficile genotypes were also analyzed phylogenetically to contextualize findings in RT078; these were ST35(046) (*n* = 34), ST54(012) (*n* = 54), ST37(017) (*n* = 64), and nontoxigenic ST26(140) (*n* = 65) (Table S2). These four genotypes underwent detailed study because they had the highest *tetM* prevalence after RT078. The isolates came from the Oxfordshire EIA-positive and EIA-negative, Oxfordshire Infant, and Leeds region isolate collections described above (8, 29). ST26(140) lacks the toxin-encoding pathogenicity locus and is therefore carried asymptomatically, providing a naturally occurring “control” for the impact of antimicrobial selection in the absence of disease.

### Genome assemblies.

C. difficile genomes were assembled from short reads generated using Illumina technology (64). Reference-based assemblies were made for genomes belonging to C. difficile clade 5 (i.e., RT078 and close relatives) as described previously (65) by mapping reads to the C. difficile M120 reference genome (66) and for non-clade 5 genomes by mapping reads to the CD630 reference genome (GenBank accession no. AM180355.1) (66) (clades defined as described in reference 35). *De novo* assembly was performed using Velvet (version 1.0.7–1.0.18) (85) and VelvetOptimiser 2.1.7 (67), optimizing kmer size (k), expected coverage (average kmer coverage of contigs), and coverage cutoff (kmer coverage threshold) to achieve the highest assembly *N*_50_ value (length of the smallest contig such that all contigs of that length or less formed half of the final assembly). Reads for unassembled genomes have been submitted to NCBI under BioProject identifier (ID) no. PRJNA304087 (8) and PRJNA381384 for the Scottish isolates (accession numbers are provided in Tables S1 and S2).

### Identification of antimicrobial resistance determinants.

The *de novo* assemblies were queried using the BLAST function of BIGSdb (68) to determine whether genes or nonsynonymous point mutations known to confer resistance to antimicrobials (including fluoroquinolones, tetracyclines, clindamycin, and aminoglycosides) were present and to extract the sequences of interest for further analysis. A list of the resistance gene sequences used to perform the BLAST search (and of their GenBank accession numbers) is provided (Table 1 and 2). For acquired resistance genes, a minimum level of 90% nucleotide sequence identity and gene coverage was required. Each unique *tetM* allele was assigned a number (allele nucleotide sequences are available at https://pubmlst.org/bigsdb?db=pubmlst_cdifficile_seqdef&page=downloadAlleles).

### Definition of multilocus sequence types (MLST).

Allele sequences used in the C. difficile MLST scheme (69) were extracted from the *de novo* assemblies using the BLAST function of BIGSdb (68). Sequence types (STs) were assigned by querying the MLST database (https://pubmlst.org/cdifficile/).

### Phylogenetic analyses.

Phylogenetic trees were built on the basis of the assemblies mapped to C. difficile ST11 reference M120 using the maximum likelihood approach implemented in PhyML version 3.1.17 (with a generalized time-reversible substitution model and the “BEST” tree topology search algorithm) (70). The trees were then corrected to account for recombination events using ClonalFrameML (71) version 1.11 (with default settings). The nodes of the trees were dated using the previously estimated C. difficile evolutionary rates of 1.1 mutation per year (30) for clade 5 STs (including RT078) and of 1.4 mutation per year for all other genotypes (72). The main period of particular interest (from 1990 to 2015) was allocated the greatest amount of horizontal space in graphical tree representations by compressing the pre-1990 period, making the trees directly comparable with respect to the post-1990 period. Events before 1990 are not shown since dating older nodes using a short-term evolutionary rate is problematic due to the time dependency of evolutionary rates (73). Graphical representations of trees were made using FigTree version 1.4.2 (74).

A quantitative assessment of clonal expansion(s) within a given phylogeny was performed as described previously (39) and as implemented previously (8). The evolutionary distinctiveness (ED) score of each isolate was calculated; the ED score was defined as being equal to the sum, for all branches on the path from the root to the leaf (isolate), of the lengths of the branches divided by the number of leaves that they support (39). For a given isolate, a low ED score indicated the presence of close relatives in the tree, whereas a high ED score indicated their relative absence. ED scores were compared across various factors using quantile regression statistics analyses, performed using Stata version 14.1 (College Station, TX, USA).

### Data availability.

Reads for unassembled genomes have been submitted to NCBI under BioProject ID numbers PRJNA304087 (8) and PRJNA381384 for the Scottish isolates (accession numbers are provided in Tables S1 and S2).
